# Sex‐dependent acrolein sensitivity in mice is associated with differential lung cell, protein, and transcript changes

**DOI:** 10.14814/phy2.14997

**Published:** 2021-10-04

**Authors:** Kiflai Bein, Rahel L. Birru, Heather Wells, Theodore P. Larkin, Tengziyi Ge, George D. Leikauf

**Affiliations:** ^1^ Department of Environmental and Occupational Health Graduate School of Public Health University of Pittsburgh Pittsburgh Pennsylvania USA

**Keywords:** acrolein, acute respiratory distress syndrome, NETs, RNAseq

## Abstract

Acrolein is a reactive inhalation hazard. Acrolein’s initial interaction, which in itself can be function‐altering, is followed by time‐dependent cascade of complex cellular and pulmonary responses that dictate the severity of the injury. To investigate the pathophysiological progression of sex‐dependent acrolein‐induced acute lung injury, C57BL/6J mice were exposed for 30 min to sublethal, but toxic, and lethal acrolein. Male mice were more sensitive than female mice. Acrolein of 50 ppm was sublethal to female but lethal to male mice, and 75 ppm was lethal to female mice. Lethal and sublethal acrolein exposure decreased bronchoalveolar lavage (BAL) total cell number at 3 h after exposure. The cell number decrease was followed by progressive total cell and neutrophil number and protein increases. The BAL total cell number in female mice exposed to a sublethal, but not lethal dose, returned to control levels at 16 h. In contrast, BAL protein content and neutrophil number were higher in mice exposed to lethal compared to sublethal acrolein. RNASeq pathway analysis identified greater increased lung neutrophil, glutathione metabolism, oxidative stress responses, and CCL7 (aka MCP‐3), CXCL10 (aka IP‐10), and IL6 transcripts in males than females, whereas IL10 increased more in female than male mice. Thus, the IL6:IL10 ratio, an indicator of disease severity, was greater in males than females. Further, H3.3 histone B (H3F3B) and pro‐platelet basic protein (PPBP aka CXCL7), transcripts increased in acrolein exposed mouse BAL and plasma at 3 h, while H3F3B protein that is associated with neutrophil extracellular traps formation increased at 12 h. These results suggest that H3F3B and PPBP transcripts increase may contribute to extracellular H3F3B and PPBP proteins increase.

## Graphical abstract

To investigate the pathophysiological progression of sex‐dependent acrolein‐induced acute lung injury, C57BL/6J mice were exposed for 30 min to lethal (75 ppm, Figure a) and sublethal , but toxic (50 ppm, Figure b), acrolein . Male mice were more sensitive than female mice. Acrolein increased neutrophils, IL10:IL6 ratio, and neutrophil extracellular traps proteins in mouse lung.

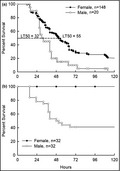

## 1 INTRODUCTION

Acute lung injury (ALI)/acute respiratory distress syndrome (ARDS) is characterized by loss of alveolar epithelial and capillary barrier function that leads to increased protein and neutrophil infiltration into the alveolar air space (Holter et al., 1986; Sinclair et al., 1994). Currently, ALI therapy is mainly limited to managed mechanical ventilation and mortality rate remains in excess of 20% (Phua et al., 2009; Wong et al., 2019). Despite extensive research in ALI during the past five decades, no standard clinical test exists to predict the severity of ALI other than the ratio of partial pressure of alveolar oxygen to the fraction of inspired oxygen (Force et al., 2012).

The etiology of ALI is complex and can be induced by direct causes such as inhaled irritants and pneumonia or indirect extrapulmonary causes such as sepsis (Reiss et al., 2012). One ALI risk factor is smoke inhalation and smoke contains several irritants including acrolein (Calfee et al., 2015; Walsh et al., 2017). Acrolein (Holter et al., 1986) is volatile at room temperature and is highly irritating to eyes and respiratory passages (Bein & Leikauf, 2011). Acrolein, as an α‐β‐unsaturated aldehyde, contains a carbonyl group and an electrophilic beta‐carbon that rapidly react with a variety of biological nucleophilic targets (Bein & Leikauf, 2020; Shao, 2011; Zarkovic et al., 2013). Acrolein can react with and adduct to the sulfhydryl of cysteine, imidazole of histidine, amino of lysine, guanidine of arginine, and deoxyguanosine of DNA (Stevens & Acrolein, 2008). The initial interaction of acrolein can alter the structure and functionality of the targeted molecule, which is followed by a cascade of complex cellular and pulmonary responses that progress with time and dictate the severity of the injury.

In ALI, mortality is influenced by sex, age, and genetic susceptibility in patient populations, which can be experimentally modeled in mice (Erickson et al., 2008; Leikauf, Concel, et al., 2011; Leikauf, Pope‐Varsalona, et al., 2010; Manzano et al., 2005; Moss & Mannino, 1996; Prows, McDowell, et al., 2003; Prows, Shertzer, et al., 1997). Despite the extensive knowledge gained about the causes, risk factors, and biology of ALI, effective clinical pharmacotherapeutic practices and molecular markers are lacking. To develop effective therapeutic interventions, more studies are needed to understand the disease‐progression mechanisms underlying survival and mortality. The aim of the present work was to determine key mouse lung pathophysiological responses elicited after acrolein exposure. Reactions between acrolein and target proteins are complete within 3 h after exposure (Bein et al., 2020). However, the onset of lethality due to acrolein exposure can be delayed by 24 h. Thus, modeling ALI with acrolein in mice provides the capacity to investigate pathophysiological responses key to the ALI progression that leads to mortality. In this study, time–course analysis was used to assess events that occur between acrolein exposure and the onset of lethality.

## 2 METHODS

### 2.1 Mouse exposure and acrolein vapor generation

This study was performed in accordance with the Institutional Animal Care and Use Committee of the University of Pittsburgh (Pittsburgh, PA, USA). Nine‐week‐old C57BL/6J mice (Jackson Laboratories) were housed under pathogen‐free conditions. Following 5 days of acclimation, mice were exposed to filtered air (control) or acrolein (50 or 75 ppm, 30 min). Acrolein vapor was generated by passing from a high‐pressure cylinder breathing air (157 ml/min for 50 ppm or 219 ml/min for 75 ppm) over liquid acrolein (Polysciences, Inc.) and introduced into a 0.32‐m^3^ stainless steel chamber. The acrolein concentration was continuously monitored using a Chemgard infrared monitor (MSA). After acrolein exposure, mouse survival time was recorded hourly during the day for the first 72 h and thereafter once every 3 h. Median lethal time (LT_50_) was calculated using logit‐probit analysis.

### 2.2 Bronchoalveolar lavage collection and analysis

At various times after acrolein exposure (1–24 h), mice were euthanized by intraperitoneally injecting pentobarbital sodium followed by severing the posterior abdominal aorta. To obtain bronchoalveolar lavage (BAL), the diaphragm was punctured, a cannula was inserted in the trachea, and the lungs were lavaged five times in series with one 0.8 ml and four 0.5 ml of phosphate‐buffered saline (PBS) containing 0.4 mM ethylenediaminetetraacetic acid (EDTA). The collected lavage was centrifuged (500 *g*, 10 min, 4°C) to pellet cells. The cell pellet was resuspended in 0.2 ml of Hank’s balanced salt solution containing 2% fetal bovine serum. Total BAL cell number was determined using a hemocytometer. To perform differential cell counts, cells were cytospun onto microscope slides and stained using Hema stain (Thermo Fisher Scientific). BAL protein concentration of the supernatant was determined using BCA protein assay and bovine serum albumin (BSA) as a standard (Thermo Fisher Scientific).

### 2.3 Mouse plasma preparation

Blood samples collected via cardiac puncture were placed in 0.5 M EDTA/saline coated microcentrifuge tubes. The plasma was separated by centrifugation (2000 *g*, 10 min) and stored (−70℃) for later Western blotting analysis.

### 2.4 Lung histology

Lungs were collected 16 h after exposure. Lungs were fixed by tracheal instillation of 10% neutralized formalin in PBS and stored overnight in the same solution. After 24 h, the storage solution was removed and washed three times with PBS. For histological examination by microscopy, lungs were paraffin‐embedded, cut into 5‐µm sections, and stained with hematoxylin and eosin. Histological scoring of lung injury was determined in ≥40 20× field/mouse lung by the Matute‐Bello et al. (2008) method as modified by Aeffner et al. (2015). This method weights neutrophils in the alveolar space (20×), neutrophils in interstitial space (14×), proteinaceous debris filling the airspaces (7×), and alveolar septal thickening (2×).

### 2.5 RNA sequencing

Total cellular RNA was isolated from control (*n* = 6 lungs/sex group) and acrolein exposed (*n* = 8 lungs/sex group) mice (*n* = 28 mice) 6 h after exposure (30 min, 50 ppm) using TRI Reagent. mRNA, enriched using oligo(dT) beads, was randomly fragmented and used for cDNA synthesis using random hexamer primers. To construct the library, the double‐stranded cDNA was end‐repaired, poly‐A‐tailed, ligated to sequencing adapters, and subjected to HiSeq Illumina X Ten sequencing by Novogene Co. Ltd. The data generated were mapped to *Mus musculus* (GRCm38/mm10) reference genome. Normalized read counts and differentially expressed genes (DEGs) were obtained using DESeq2 software (Anders & Huber, 2010). Estimated p value was obtained by negative binomial distribution modeling method followed by Bonferroni corrected for multiple comparisons and the resulting adjusted *p* < 0.05 was considered significant. Kyoto Encyclopedia of Genes and Genomes (KEGG) murine pathways, Gene Ontology categories of Biological Process and Molecular Function, and LINCS L1000 Analysis 9 were assessed using Enrichr (Xie et al., 2021) and an adjusted *p* < 0.05 was considered statistically significant.

### 2.6 C57BL/6J lung transcript analyses

Total cellular RNA was isolated from control and acrolein exposed mouse lungs using TRI Reagent and quantified by *A*_260_ absorbance determination (Take3; BioTek Instruments). The mRNA was analyzed by quantitative real‐time polymerase chain reaction (qRT‐PCR). To quantify H3F3B and PPBP transcripts by qRT‐PCR, DNase I‐treated RNA (0.9 µg) was reverse‐transcribed using an iScript cDNA synthesis system in a 30 µl reaction volume and diluted 5× using RNase‐free water. An aliquot of the cDNA synthesis product (2 µl) was used in a subsequent qRT‐PCR analysis (Cat. No. 4369016, TaqMan Gene Expression Master Mix; Applied Biosystems [ABI]). The qRT‐PCR analysis was performed with a 7900HT Fast Real‐Time PCR System using the following conditions: 50°C for 2 min, 95°C for 10 min followed by 40 cycles of 95°C for 15 s and 60°C for 1 min. The primers used were the internal control ribosomal protein L32 (RPL32, ABI Cat. No. Mm02528467_g1), H3F3B (ABI, Cat. No. Mm00787223_s1), and PPBP (ABI, Cat. No. Mm00470163_m1). The comparative cycle number threshold (*C*_t_) method (∆∆*C*_t_) was used to determine transcript change.

### 2.7 Sodium dodecyl sulfate‐polyacrylamide gel electrophoresis and Western blot analysis

To detect proteins, samples were incubated in Laemmli’s sodium dodecyl sulfate (SDS)‐sample buffer (95℃, 5 min) and separated using 4%–20% mini‐protean TGX gels (Cat. No. 456–1094; Bio‐Rad). For albumin, immunoglobulin G (IgG), hemopexin, and haptoglobin detection (10 µl/lane of BAL, 0.5 µl/lane of plasma), SDS‐polyacrylamide gel electrophoresis‐separated proteins were transferred to polyvinylidene difluoride membrane (Thermo Fisher Scientific; Cat. No. 88518). The membrane was rinsed twice (5 min each) in Tris‐buffered saline (TBS; 20 mM Tris, 500 mM NaCl, pH 7.5) and blocked for 1 h with 5% nonfat milk or BSA in TBS. The membrane was then incubated with goat anti‐mouse albumin [Immunology Consultants Laboratory [ICL]; Cat. No. GAL‐90A, 1/5000], horseradish peroxidase (HRP)‐conjugated anti‐mouse IgG (R&D Systems; Cat. No. HAF007, 1/5000), rat anti‐mouse hemopexin (R&D Systems; Cat. No. MAB7007, 1/1000) or goat anti‐mouse haptoglobin (ICL; Cat. No. 90A‐Z, 1/1000) antibody diluted in 1% BSA in TBS containing 0.1% Tween 20 (TBST) for 1–2 h (23℃). For H3.3 histone B (H3F3B) and pro‐platelet basic protein (PPBP, aka CXCL7) detection, proteins (25–35 µl/lane of BAL, 1 or 10 µl/lane of plasma) were separated using 4%–20% mini‐protean TGX stain‐free gels (Bio‐Rad Laboratories; Cat. No. 4568094) and were transferred to immobilon membrane (Thermo Fisher Scientific; Cat. No. ISEQ00005) in sodium carbonate transfer buffer (Rossmann & Stillman, 2013). The immobilon membrane blot was briefly rinsed in water, dried at 37°C for 1 h, and prewet in methanol. The membrane was rinsed twice (5 min each) in TBS and blocked for 1 h (23℃) with 5% BSA in TBS. The membrane was then incubated with rabbit anti‐histone H3 (Abcam; Cat. No. AB1791, 1/3000) or rabbit anti‐mouse CXCL7 antibodies (Abcam; Cat. No. 206406, 1/2000). To apply secondary antibody, the membrane was rinsed and washed twice in TBST (10 min each), and then incubated for 1 h (23℃) in HRP‐conjugated goat anti‐rabbit IgG (Cell Signaling Technology; Cat. No. 7074, 1/20,000), mouse anti‐goat IgG (Thermo Fisher Scientific; Cat. No. 31400, 1/10,000) or goat anti‐rat IgG (Cell Signaling Technology; Cat. No. 7077, 1/3000) diluted in 1% BSA in TBST. After three to four washes, the immunoblot was incubated for 1–3 min in SuperSignal West Pico chemiluminescent substrate (Thermo Fisher Scientific; Cat. No. 34080) and the protein signal developed after exposure to x‐ray film (Phenix Research Products; Cat. No. F‐BX57).

### 2.8 Statistical analysis

Statistical analysis was performed and significance (*p* ≤ 0.05) determined by Holm–Sidak all pairwise multiple comparison procedures (SigmaStat Program; SPSS, Inc.).

## 3 RESULTS

### 3.1 Determination of lethal and sublethal acrolein doses in mice

**FIGURE 1:**
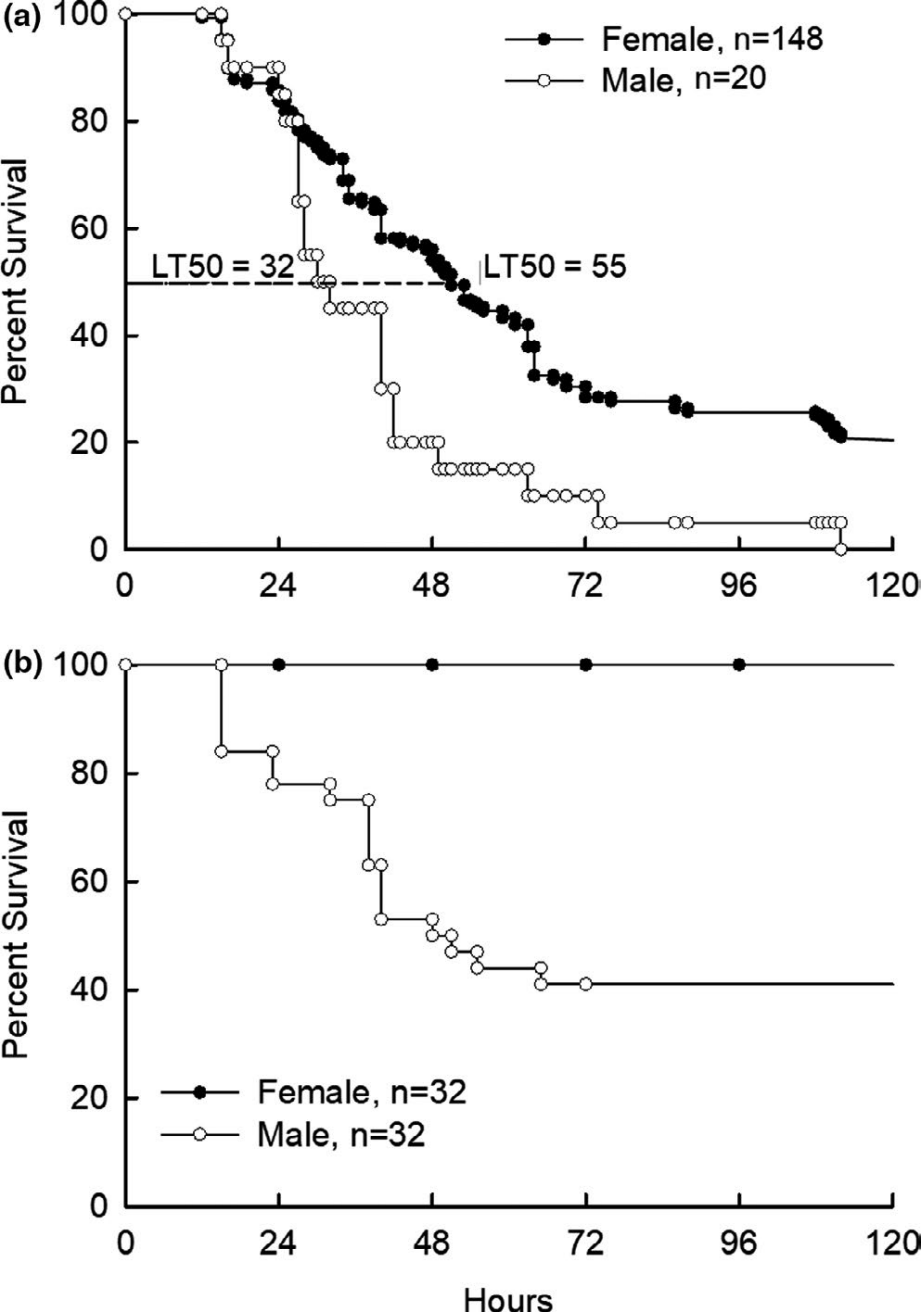
Survival of C57BL/6J mice following acrolein exposure. Survival of female and male C57BL/6J mice exposed to (a) 75 ppm or (b) 50 ppm acrolein for 30 min and returned to filtered air and survival monitored for up to 120 h. At either concentration, male C57BL/6J mice are more sensitive to acrolein exposure compared with female mice as determined by Kaplan–Meier log‐rank survival (*p* < 0.001)

Previously, Conklin et al. (2016) reported that male C57BL6 mice were more sensitive to inhaled acrolein as compared to female mice. Because male mice are more susceptible, we sought to contrast the response of male with female mice after exposure to sublethal and lethal acrolein doses to investigate the pathophysiological events that contribute to this difference. Male and female C57BL/6J mice were exposed to 75 or 50 ppm acrolein for 30 min. Exposure to 75 ppm acrolein resulted in 100% male and 79% female mice mortality within 120 h after exposure (Figure 1a). Within the first 24 h after acrolein exposure, mortality minimally occurred in male and female mice. With time, however, the survival curves for male and female mice diverged. The median lethality time (LT_50_) for male mice (LT_50_ = 33 h) was shorter than that of female mice (LT_50_ = 55 h). Exposure to 50 ppm acrolein resulted in 60% male and 0% female lethality within 120 h after exposure (Figure 1b).

**FIGURE 2:**
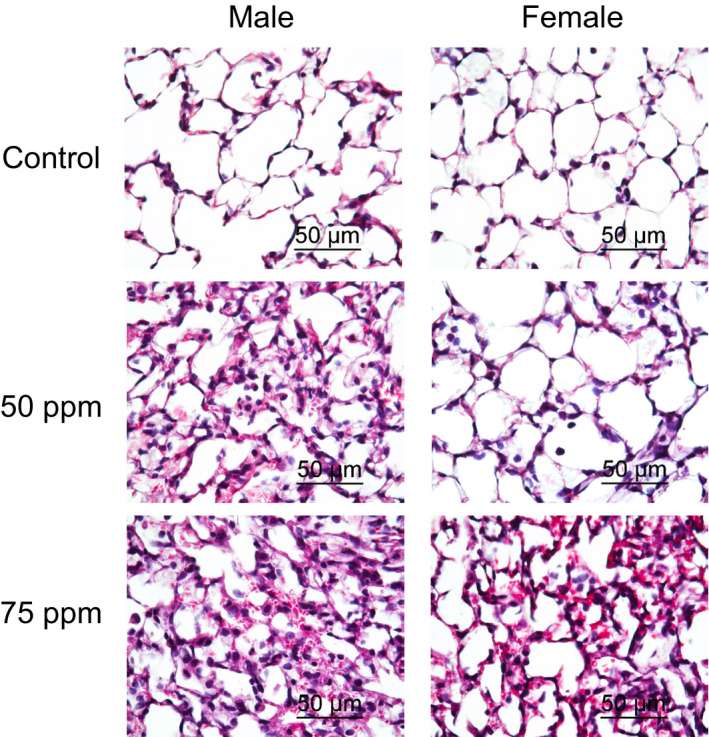
Lung histology of C57BL/6J mice following acrolein exposure. Acrolein exposure increased alveolar wall thickening, lung proteinaceous deposits, and leukocyte infiltrates. Male (Left panels) and female (Right panels) mice were exposed to filtered air (Top panels), 50 ppm (Middle panels), or 75 ppm (Bottom panels) acrolein for 30 min and lungs collected 16 h after exposure for hematoxylin and eosin staining

Consistent with these findings, histological evidence of lung injury was more evident in males as compared to females following exposure to 50 ppm, while at 75 ppm lung injury was comparable in males and females (Figure 2). In male and female mice exposed to filtered air control, the alveolar lumen was clear, neutrophils were not observed in the alveolar space and few in the interstitial space, proteinaceous debris, and alveolar wall thickening were absent. The control histological score was 0.015 + 0.006 and 0.018 + 0.008 (mean + standard error of mean [SEM]) for males and females, respectively. In male mice exposed to 50 or 75 ppm, the histological score increased to 0.274 + 0.015 (*p* < 0.01) and 0.346 + 0.027 (*p* < 0.01), respectively. In female mice exposed to 50 ppm, the histological score was 0.043 + 0.023 (*p* = 0.27), which was not significantly different from the control. In females exposed to 75 ppm, the histological score increased to 0.271 + 0.043 (*p* < 0.01). Thus, 50 ppm acrolein concentration was selected as a dose that was lethal to male but sublethal to female C57BL/6J mice.

**FIGURE 3:**
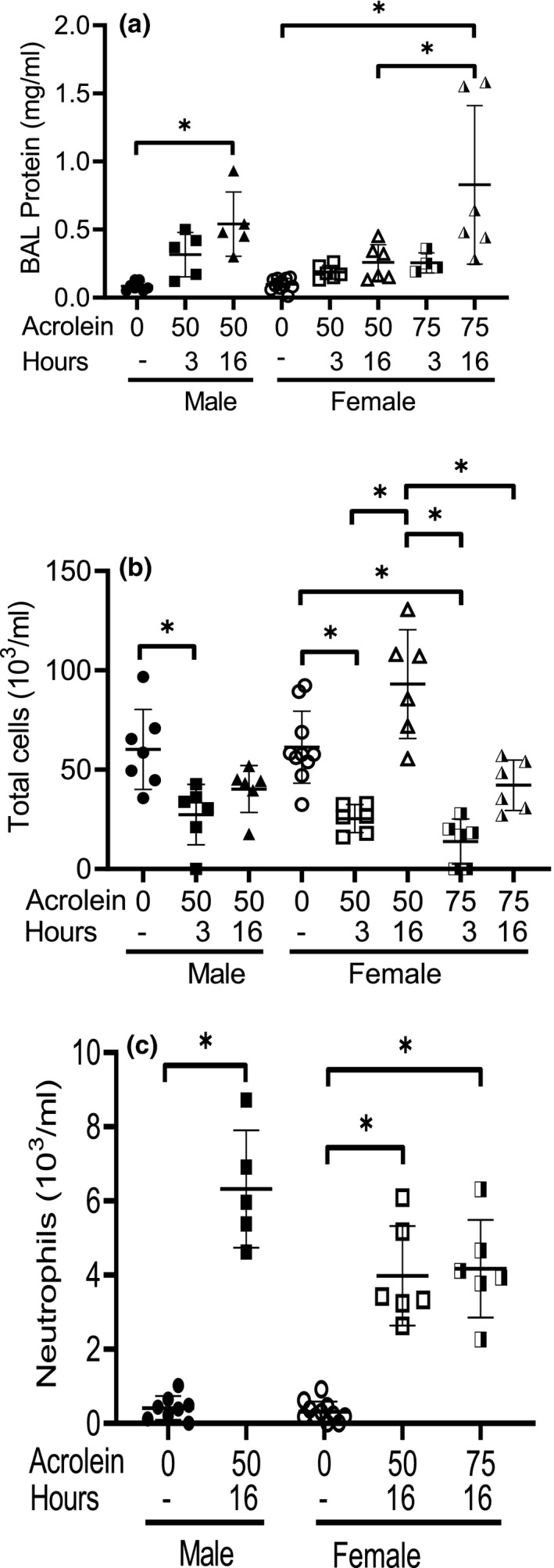
Bronchoalveolar (BAL) protein, total cells, and neutrophils in C57BL/6J mice exposed to 50 or 75 ppm acrolein for 30 min. BAL was collected from filtered air exposed male and female mice (0), male mice exposed to 50 ppm, or female mice exposed to 50 or 75 ppm acrolein for 30 min. Following exposure, mice were returned to filtered air and BAL was collected 3 or 16 h after exposure. BAL (a) protein, (b) total cells, and (c) neutrophils were determined. Each symbol represents one mouse and midline indicates mean and bars indicate standard deviation (SD, *n* = 5–9 mice/group). *Values are significantly different (*p* < 0.01) as determined by one‐way analysis of variance with Holm–Sidak all pairwise multiple comparison procedure

To assess the progression of lung injury, BAL was performed at 3 and 16 h after exposure in male and female mice exposed to 50 ppm or female mice exposed to 75 ppm acrolein (Figure 3). The 3 h time was selected because it is the time when initial acrolein reactions with targets are complete and 16 h was selected because it is typically the time when inflammation is at its peak. At 16 h after exposure, BAL protein increased in male mice exposed to 50 ppm acrolein compared to control male mice whereas BAL protein in female mice exposed to 50 ppm acrolein did not increase compared with control female mice (Figure 3a). BAL protein also increased in female mice exposed to 75 ppm acrolein 16 h after exposure compared to female mice exposed to 50 ppm acrolein. Increased BAL protein was evident, at least in some mice, by 3 h after exposure. These observations suggest that acrolein‐induced increased BAL protein reflects the phenotypic difference in acrolein‐induced sublethal compared to lethal sensitivity;

At 3 h after exposure, BAL total cell number, which is predominantly (~99%) macrophages, decreased in male and female mice exposed to 50 ppm acrolein as well as female mice exposed to 75 ppm acrolein (Figure 3b). Interestingly, BAL total cell number increased at 16 h post‐exposure in female mice exposed to 50 ppm acrolein and was higher than the increase observed in male mice exposed to 50 ppm acrolein or in female mice exposed to 75 ppm acrolein (Figure 3b). The neutrophil percentage, which was <1% in control male and female mice, increased in acrolein exposed mice to 14% in male mice exposed to 50 ppm acrolein, 4% in female mice exposed to 50 ppm acrolein, and 10% in female mice exposed to 75 ppm acrolein. Because the total cell number recovered in female mice exposed to 50 ppm acrolein was higher, the neutrophil number in female mice exposed to 50 and 75 ppm acrolein was comparable (Figure 3c). Nonetheless, it should be noted that the ratio of total BAL cells to neutrophils was higher in female mice exposed to 50 ppm acrolein compared with male mice exposed to 50 ppm acrolein and female mice exposed to 75 ppm acrolein. These findings suggest that during the early phase of ALI in mice, the decreased total cell number is followed by increased protein and neutrophils.

### 3.2 Baseline lung transcripts that differ between male and female C57BL/6J mice

Lung mRNA from male and female mice exposed to filtered air (control *n* = 6 mice/sex) was analyzed by RNASeq. Compared to control female mice, control male mice had 88 increased and 66 decreased transcripts (Table S1, https://figshare.com/s/10.6084/m9.figshare.14454309). Of these, 14 increased and three decreased by more than 1.5‐fold. Because little is known about sexual dimorphisms of constitutive mRNA in the lung, transcripts with related functions with an adjusted *p* value < 0.05 but below a threshold of 1.5‐fold are included for this analysis.

The greatest increased transcripts in control males as compared to control females were DEAD (Asp–Glu–Ala–Asp) box polypeptide 3 Y‐linked (DDX3Y), eukaryotic translation initiation factor 2 subunit 3 structural gene Y‐linked (EIF2S3), lysine (K)‐specific demethylase 5D (KDM5D), and ubiquitously transcribed tetratricopeptide repeat gene Y chromosome (UTY), which are products of genes located on the Y‐chromosome. Male mice had corresponding decreased DDX3X, EIF2S3X, KDM5C, KDM6A, which are products of X‐chromosome genes that escape X inactivation. Interestingly, DDX3X is critical to innate immunity and coordinates cell stress granule and the NLRP3 inflammasome functions.

Other sexually dimorphic transcripts increased in male as compared to females included 5‐aminolevulinate synthase 2 (ALAS2), which catalyzes the first step of heme biosynthesis and the hemoglobin genes, HBA‐A1, HBB‐BS, and HBB‐BT. Complement component 3 (C3) and C7 increased in male as compared to female mice. Similarly, xenobiotic metabolism transcripts carboxylesterase 1F (CES1F), CES1G, metallothionein 2 (MT2), cytochrome P450 family 2 subfamily e polypeptide 1 (CYP2E1), NAD(P)H dehydrogenase quinone 1 (NQO1), and aldehyde dehydrogenase family 1 subfamily A2 (ALDH2A1) increased in male as compared to female mice. Four anti‐proteinase genes, serine (or cysteine) peptidase inhibitor clade A member 3 M (SERPINA3 M), SERPINA3N, tissue inhibitor of metalloproteinase 1 (TIMP1), and secretory leukocyte peptidase inhibitor (SLPI) were greater in male as compared to female mice.

Transcripts decreased in male as compared to female mice included eight associated with cilia including dynein axonemal heavy chain 6 (DNAH6), DNAH10, and anterior gradient 3 (AGR3). Also, noteworthy, adrenomedullin (ADM) was decreased in males as compared with females. The resulting protein has several functions including vasodilation and regulation of hormone secretion.

### 3.3 Increased DEGs pathway analysis

**FIGURE 4:**
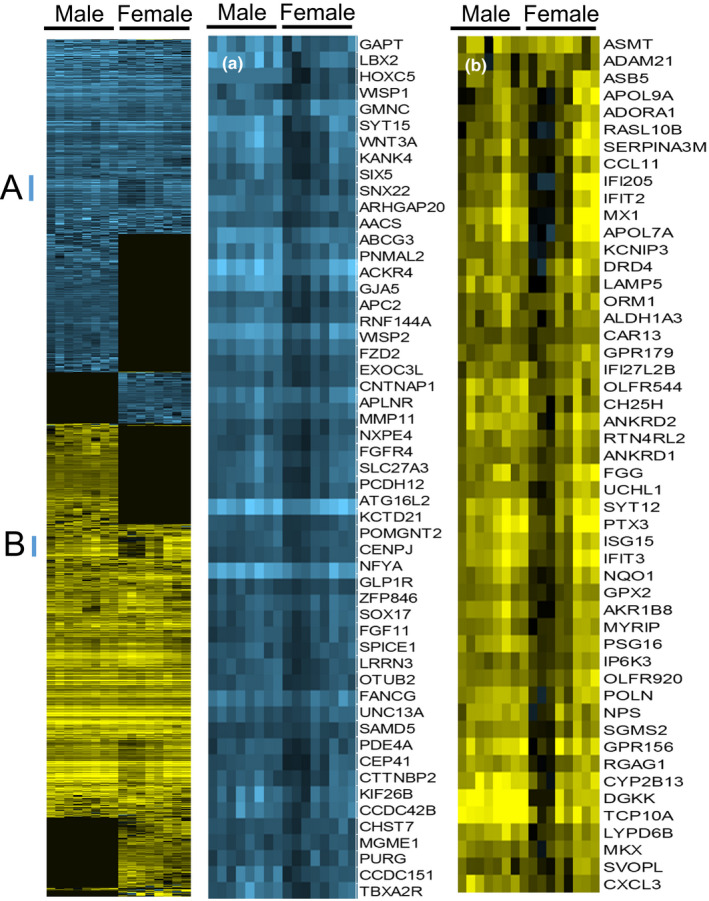
Differentially expressed genes altered in mouse lungs following acrolein exposure. Mice were exposed to air (control) (*n* = 6/sex) or 50 ppm acrolein (*n* = 8/sex) for 30 min and returned to filtered air. Lungs were collected 6 h after exposure and transcripts were analyzed by RNASeq. Transcripts that (Blue) decreased or (Yellow) increased by absolute value twofold and adjusted *p* value < 0.5 were arranged by centroid linked hierarchical clustering using Gene Cluster 3.0 and visualized by Java TreeView. (Left panel) Each column is one mouse (*n* = 8 male and 8 female) and each row is one transcript (*n* = 1991) with enlarged cluster in which female mice had an uneven (Cluster A middle panel) decreased or (Cluster B right panel) increased response as compared to male mice

An analysis of KEGG murine pathways and Gene Ontology (GO) categories of Biological Process and Molecular Function was performed comparing the transcriptional responses of male and female C57BL/6J mice following acrolein exposure (*n* = 8 mice/sex) as compared to filtered air exposure (control *n* = 6 mice/sex) (Figure 4). To identify pathways that were common in both males and females, we first identified DEGs that were increased in both male and female mice (Table S2, https://figshare.com/s/10.6084/m9.figshare.14454321). Common to both males and females were 4050 protein‐coding DEGs of which 3787 had known function and were used for pathway analysis. The total number of enriched KEGG murine pathways equaled 129 pathways that included several stress response pathways, for example, MAPK, IL‐17, and tumor necrosis factor (TNF) signaling pathways, as well as ubiquitin‐proteasome system‐related proteins, that is, chaperones, ubiquitin ligases, and proteasome components. The most enriched GO Biological Process was Protein modification by small protein removal (GO:0070646) and the most enriched GO Molecular Function was RNA binding (GO:0003723).

In the results that follow, transcripts that differed either in males or in females were used to identify pathways and ontologies (with exemplar transcript symbols presented in parenthesis). We identified pathways for the 671 DEGs of which 609 had known functions that increased only in males (Table S3, https://figshare.com/s/10.6084/m9.figshare.14454312). No KEGG murine pathway was enriched. The only enriched GO Biological Process was Maturation of SSU‐rRNA (GO:0030490) (adjusted *p* = 0.02). No GO Molecular Function ontology was enriched.

**FIGURE 5:**
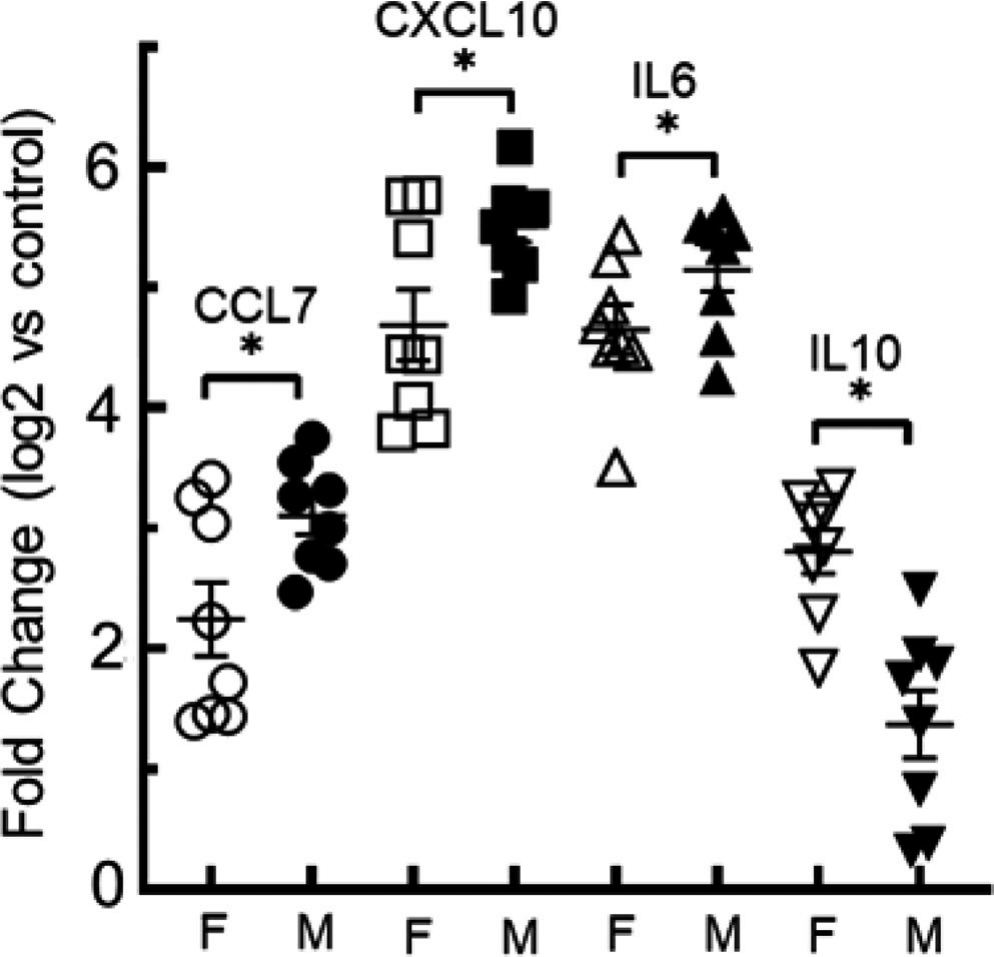
Lung cytokines altered in male compared to female mice following acrolein exposure. Mice were exposed to air (control) (*n* = 6/sex) or 50 ppm acrolein (*n* = 8/sex) for 30 min and returned to filtered air. Lungs were collected 6 h after exposure and transcripts were analyzed by RNASeq. Chemokine (C–X–C motif) ligand 7 (CCL7 aka MCP‐3), chemokine (C–C motif aka IP‐10) ligand 10 (CXCL10), interleukin 6 (IL6), increased more in male as compared to female mice. In contrast, IL10 increased more in female as compared to male mice. *Significantly different between sexes as determined by analysis of variance (ANOVA) followed by one‐way analysis of variance with Holm–Sidak all pairwise multiple comparison procedure

Following acrolein exposure, 252 DEGs increased more in males than females of which 239 had known function and included increased myeloperoxidase (1.10 ± 0.43 log2 fold adj. *p* < 0.04) and neutrophilic granule protein (2.28 ± 0.31 log2 fold adj. *p* < 1.1E − 06). Neither transcript was significantly altered in female mice (adj. *p* > 0.05). These indicators suggest an increase in neutrophil infiltration in male mice. In recent studies in humans, profiling studies have identified MCP‐3 (CCL7), IP‐10 (CXCL10), IL‐6 (IL6), and other cytokines as predictors for the progression and severity of ALI, which increased more in males than females (McElvaney et al., 2020; Yang et al., 2020). We, therefore, compared the lung transcripts of CCL7, CXCL10, and IL6 in male and female mice. Each increased more in male than female mice (Figure 5). In contrast, IL10 increased more in female mice. Thus, the IL6:IL10 ratio was 5.82 ± 1.69 for males and 1.68 ± 0.06 for females (mean ± SEM, *p* < 0.05).

The DEGs that increased more in male than female mice were enriched in 12 KEGG pathways including Glutathione metabolism (GCLC, GCLM, GPX2, GSR, GSS, GSTA1, A2, and O1), Ferroptosis (FTL1, FTH1, SLC3A2, and SLC7A11), and Metabolism of xenobiotics by cytochrome P450 (ALDH1A3, ALDH3A1, CBR3, CYP1B1, and GSTs) (Table S4, https://figshare.com/s/10.6084/m9.figshare.14454306). Thirty‐two GO Biological Process pathways were enriched including Cellular response to oxidative stress (GO:0034599) and Glutathione metabolic process (GO:0006749) and included increased transcripts in the Glutathione metabolism KEGG pathway. Similarly, the single enriched GO Molecular Function ontology was Glutathione transferase activity (GO:0004364).

The 1217 increased DEGs occurring only in females contained 1123 protein‐coding DEGs with known function (Table S5, https://figshare.com/s/10.6084/m9.figshare.14454318). The enriched 26 KEGG murine pathways included Oxidative phosphorylation (ATP1A4, COX10, and NDUFA10), Phagosome (CALR, COMP, FCGR4, H2‐D1, ITGAM, MRC1, and SCARB1), Lysosome (ACP5, ARSB, CTSK, CTSW, LAPTM5, and SLC11A1), and Natural killer cell‐mediated cytotoxicity (H2‐K1, IFNAB, KLRA4, KLRA7, KLRC1, KLRC2, KLRK1, NFATC2, PRF1, and ZAP70). The most enriched of 74 enriched GO Biological Process was Extracellular matrix organization (GO:0030198) (A2 M, COL4A5, FN1, HSPG2, ITGA4, LUM, and TNC) and most enriched of 14 enriched GO Molecular Function was Integrin binding (GO:0005178) (ACTN2, ADAMTS8, ANGPTL3, LTBP4, P4HB, VCAM1).

Differentially expressed genes increased more in females as compared to males numbered 376 of which 358 had known function (Table S6, https://figshare.com/s/10.6084/m9.figshare.14456352). These DEGs were enriched in 44 KEGG pathways including T cell receptor signaling pathway (CD3D, CD28 CTLA4, ICOS, ITK, LAT, and PDCD1) and Cell adhesion molecules (CAMs) (CD6, CDH15, CLDN 22, and SDC4). The enriched GO Biological Process pathways numbered 105 and included Cellular response to cytokine stimulus (GO:0071345) (CCL4, CCL5, CXCL1, CSF3, and CSF3R). The enriched GO Molecular Function included Cytokine activity (GO:0005125) (CXCL16, IL10, and OSM).

### 3.4 Decreased DEGs pathway analysis

Pathways that were common to males and females contained 3483 DEGs with a known function that were decreased both in male and female mice (Table S7, https://figshare.com/s/10.6084/m9.figshare.14454315). Four KEGG pathways were enriched and included Peroxisome (ABCD1, ACOX3, ACSL3, CROT, DDO, DECR2, MVK, NUDT7, PEX6, 10, and 26, and PXMP2 and 4) and Fanconi anemia pathway (FANCA, B, C, F, G, and L, MH1, and TELO2). Twelve GO Biological Process ontologies were enriched and included cilium assembly (GO:0060271) (ATAT1, BBS1, 7, and 10, CC2D2A, CENPJ, DNAAF1, FOXJ1, MKKS, RSPH4A, VANGL2, 10 CEP, and 9 IFT transcripts), and DNA repair (GO:0006281) (ALKBH2, CHEK2. DDB2, DNA2, ENDOV, ERCC2 and 3, GTF2H2, 3, and 4, RAD9A, 50, 51, 52, and 9A, RFC2, WRN, XRCC1, and 9 POL transcripts). No GO Molecular Function was enriched significantly.

The 797 decreased DEGs in only male mice included 713 transcripts with known function. The three enriched KEGG murine pathways included Protein digestion and absorption (ATP1A3, PRCP, and SLC16A10) (Table S8, https://figshare.com/s/10.6084/m9.figshare.14454324). No GO Biological Process or Molecular Function ontology was enriched. Following acrolein exposure, 560 DEGs with 555 of known function decreased more in males as compared to females. Three KEGG pathways that were enriched in decreased DEGs included Wnt signaling pathway (FZD2, WNT2, WNT3A, WNT7A, VANGL2) and Cytokine‐cytokine receptor interaction (CCR2, CSF2, CX3CR1, CXCR311, IL27RA, IL9R, and TNFSF10 and 13) (Table S9, https://figshare.com/s/10.6084/m9.figshare.14454327). No GO Biological Function ontologies were enriched and three GO Molecular Function ontologies included death receptor activity (GO:0005035) (CD27, EDA2R, NGFR, and TNFRSF1B, 8, and 14).

Differentially expressed genes decreased only in females equaled 993 of which 876 had known function. No enrichment was present in KEGG pathways or GO Molecular Function pathway. Two GO Biological Processes included cilium movement (GO:0003341) (ARMC4, GAS8, and DNAH3, 9, and 11) and nucleotide‐excision repair (GO:0006289) (ERCC1, ERCC6, POLD3, R2B. R2F, and R2J) (Table S10, https://figshare.com/s/10.6084/m9.figshare.14456355). Similarly, 164 DEGs with 145 of known function that decreased more in females than in males did not reveal any enrichment in KEGG pathways. The decreased transcripts were enriched in one GO Biological Process ontology: cilium organization (GO:0044782) (CATIP, DNAH5, and DNAH6) and two GO Molecular Function ontologies including ATP‐dependent microtubule motor activity (GO:1990939) (KIF27, and DNAH5, 6, and 10) (Table S11, https://figshare.com/s/10.6084/m9.figshare.14456358).

### 3.5 H3.3 histone B (H3F3B) and PPBP transcripts and protein increased during ALI

**FIGURE 6:**
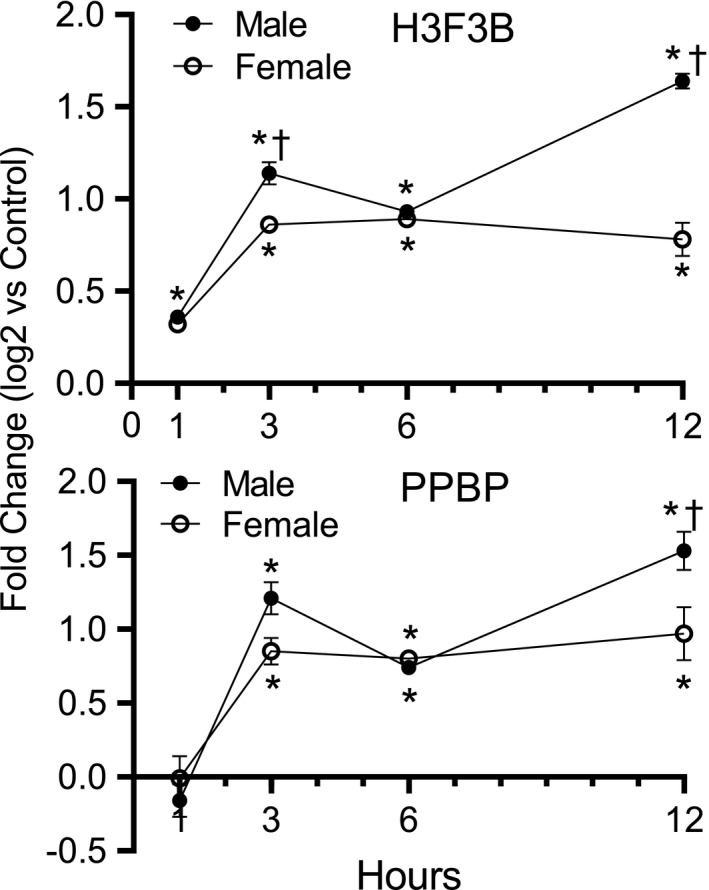
Lung H3.3 histone B (H3F3B) and pro‐platelet basic protein (PPBP) transcripts increased in acrolein exposed male and female C57BL/6J mice. Mice were exposed to air (control) or 50 ppm acrolein for 30 min and returned to filtered air. Lungs were collected 1, 3, 6, and 12 h after exposure. H3F3B and PPBP transcripts were analyzed by quantitative real‐time polymerase chain reaction (qRT‐PCR). H3F3B (Top panel) and PPBP (Bottom panel) transcripts increased by 3 h after exposure and remained elevated up to 12 h after exposure compared to control. (Values are Mean ± standard error of means [SEM], *n* = 6 mice/group). *Values are increased compared to control, ^†^increased in female compared to male (*p* < 0.01) as determined by one‐way analysis of variance (ANOVA) with Holm–Sidak all pairwise multiple comparison procedure

A key difference between males and females was increased neutrophil in male mice as indicated by BAL and transcriptome analyses. Increased neutrophils in the lung can lead to extracellular traps consisting of histones, DNA, and platelet aggregates, which are an indication of the severity of lung injury (Bdeir et al., 2017; Xu, Zhang, Pelayo, et al., 2009). These studies (Xu, Zhang, Pelayo, et al., 2009) demonstrated that extracellular histones mediated mortality of mice in lipopolysaccharide, TNF, or cecal ligation and puncture models of sepsis. Also, the platelet protein PPBP (aka CXCL7) mediated acid‐induced ALI (Bdeir et al., 2017). To evaluate ALI progression, lung RNA was isolated from male and female C57BL/6J control and exposed mice 1, 3, 6, and 12 h after exposure to 50 ppm acrolein. H3F3B and PPBP transcript levels were assessed by qRT‐PCR analysis (Figure 6). H3F3B and PPBP transcripts increased by 3 h after exposure in male and female mice compared to control mice. The transcript increase in acrolein exposed mice was maintained up to 12 h post‐exposure. H3F3B transcript was higher at 3 and 12 h after exposure in male mice compared to female mice. PPBP transcript was higher at 12 h after exposure in male mice compared to female mice.

**FIGURE 7:**
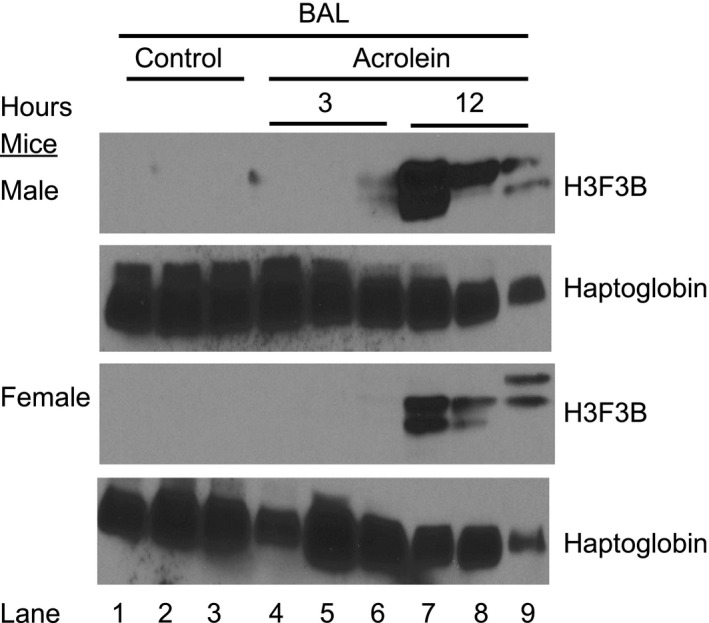
Delayed H3.3 histone B (H3F3B) protein detection in bronchoalveolar lavage (BAL) of acrolein exposed male and female C57BL/6J mice. Mice were exposed to 50 ppm acrolein for 30 min and returned to filtered air. BAL (35 µl/lane) from control or acrolein exposed mice was collected 3 or 12 h after exposure and analyzed by Western blotting for H3F3B protein. Haptoglobin was included to demonstrate protein loading

To examine the time course of H3F3B protein increase, control, 3 and 12 h BAL samples from male and female mice exposed to 50 ppm acrolein were assessed by Western blotting (Figure 7). H3F3B protein was detected in 12 h BAL from acrolein exposed mice but not in 3 h BAL samples, suggesting time‐dependent H3F3B protein accumulation. To exclude the possibility that the observed BAL H3F3B or PPBP protein increase was simply as a consequence of the increased BAL protein in acrolein exposed mice compared with control mice, plasma H3F3B and PPBP protein levels were assessed (Figure [Link], [Link], https://figshare.com/s/10.6084/m9.figshare.14946405). Albumin, IgG, and hemopexin increased and haptoglobin decreased in BAL from acrolein exposed mice compared to control mice whereas albumin, IgG, and hemopexin slightly decreased in acrolein exposed plasma samples. Following acrolein exposure, due to dysregulated alveolocapillary barrier function, hemopexin moves from the region of high concentration in the vasculature to the alveoli. In contrast, haptoglobin which is induced in the lung by acrolein crosses from the alveoli into the vasculature. Compared to control mice, H3F3B and PPBP proteins increased in both BAL and plasma samples from acrolein exposed mice. This indicates that the increased BAL H3F3B and PPBP levels are due to acrolein exposure and not simply due to increased permeability into the alveoli due to respiratory barrier dysfunction.

## 4 DISCUSSION

Exposure to high acrolein concentrations can lead to death within hours. In one case report, a 4‐year‐old boy died due to smoke inhalation from an overheated fryer (Gosselin et al., 1979). Histopathology indicated massive cellular desquamation of the bronchial lining and the cause of death was attributed to acrolein inhalation. Exposure to 10 and 150 ppm acrolein has been estimated to cause death within minutes (Einhorn, 1975; Henderson & Haggard, 1943). Although reported human fatalities due to acrolein per se are rare, acrolein is one of the toxic components of smoke (Vos et al., 2008; Dost, 1991; Eiserich et al., 1995). Inhalation injury was associated with increased mortality in burn patients (Belenkiy et al., 2014; Silva et al., 2017; Veeravagu et al., 2014).

Previously, Conklin et al. determined that 210–275 ppm acrolein (30 min) produced death within 24 h, which was lethal to male mice whereas 90% of female mice survived (Conklin et al., 2016). In our study, we determined 50 ppm for male mice and 75 ppm for female mice (30 min) produced death when monitored for 120 h. Strain differences in sensitivity to acrolein have been reported (Leikauf et al., 2011). However, in both the Conklin and our studies, C57BL/6J mice of the same strain obtained from the same supplier (Jackson Laboratory) were tested. Differences in sample size, observational frequency, exposure systems, or housing conditions may explain these differences.

Mouse lethality due to acrolein inhalation was delayed, in general, by 24 h or longer. Thus, analysis of key acrolein‐induced lung pathophysiologic responses was performed within the first 24 h after exposure. One of the early changes determined following acrolein exposure was reduced BAL cell number, including in female mice exposed to the sublethal, but toxic, dose of 50 ppm acrolein. The cell number decrease caused by acrolein exposure was followed by time‐dependent cell number increase. In female mice exposed to 50 ppm acrolein, but not in male mice exposed to 50 ppm or female mice exposed to 75 ppm acrolein, the total BAL cell number returned to control levels by 16 h after exposure.

The total cell number increase after acrolein exposure was accompanied by a neutrophil increase. The percentage of neutrophils in female mice exposed to 50 ppm acrolein was lower than the percentage of neutrophils in male mice exposed to 50 ppm acrolein and female mice exposed to 75 ppm acrolein. Previous studies reported suppression of pulmonary immune defense against bacterial and viral infection in mice subjected to prolonged acrolein exposure (Astry & Jakab, 1983; Green, 1985; Jakab, 1977, 1993; Li & Holian, 1998). The acute reduced BAL cell number is suggestive of compromised alveolar macrophage surveillance in the lungs. Improved restoration of alveolar cell number in female mice exposed to 50 ppm may contribute to the resolution of the injury and reduce lethality. The observed increase of IL10, a resolving cytokine, in female mice is consistent with this possibility. The resolution of ARDS in sepsis patients was associated with an increased number of macrophages (Steinberg et al., 1994). However, a consistent trend of a higher polymorphonuclear leukocyte concentration has been observed in patients who died compared with those who lived (Steinberg et al., 1994).

Transcriptome profiling identified several pathways that were shared by male and female mice following 50 ppm acrolein exposure. Males and females had increased Th17 responses as indicated by increased IL17C, IL17F, and S100A8. However, male mice displayed greater neutrophil, glutathione metabolism, and oxidative stress responses than females. Females displayed greater macrophage‐mediated responses, which was indicated by augmented oxidative phosphorylation, phagosome, and lysosome pathways in the RNASeq analysis. This is consistent with the return of total cells in female mice following exposure to 50 ppm measured at 16 h. Lung CCL7 (aka MCP‐3), CXCL10 (aka IP‐10), and IL6 transcripts also increased more in male compared with female mice, whereas IL10 increased more in female mice as compared with male mice. Thus, the IL6:IL10 ratio, which is an indicator of ALI disease severity (McElvaney et al., 2020), increased more in male mice than female mice following acrolein exposure. The utility of the IL6:IL10 ratio as a predictor of outcome in other forms of ALI seems to warrant further investigations. The faster recovery of BAL total cell count and increased IL10, a macrophage‐derived anti‐inflammatory cytokine, suggest that a restorative macrophage response occurs sooner in females than male mice.

Total and induced BAL protein changes occur in a time‐dependent manner in acrolein exposed mice. Increased total BAL protein was less in female mice exposed to 50 ppm acrolein compared to male mice exposed to 50 ppm acrolein and female mice exposed to 75 ppm acrolein. Increased BAL protein is likely due to the high abundance of blood proteins such as albumin and IgG leaking into the alveolar space. However, protein leak from the alveoli into the blood compartment also is implicated by increased plasma haptoglobin in acrolein exposed mice compared with control mice (Figure [Link], [Link], https://figshare.com/s/10.6084/m9.figshare.14946405).

Acrolein exposure increased H3F3B and PPBP transcripts at 3 h after acrolein exposure and the increased transcript level was maintained up to 12 h. These results suggest that H3F3B and PPBP transcript increase may contribute to extracellular H3F3B and PPBP protein increase. To the authors’ knowledge, this work is the first to report a possible link between H3F3B and PPBP transcript and protein increases in acrolein exposed mice. The BAL protein change also reflects a change in composition as exemplified by increased H3F3B and PPBP proteins in BAL of acrolein exposed mice. Increased H3F3B and PPBP proteins were detected in the delayed phase of acrolein‐induced lung injury in BAL and plasma. It is not clear whether the delayed detection of the proteins is due to sensitivity limitation of the detection system, delayed appearance of the proteins in the extracellular compartment, or low protein concentration. PPBP protein was also detected in control mouse plasma. Platelet activation due to inherent technical manipulation during blood collection or thereafter cannot be ruled out. Multiple H3F3B protein bands were detected by Western blotting, the faster migrating forms likely being cleaved H3F3B protein species (Nair et al., 2018). No protein signal was obtained when antibodies against H1, H2 or H4 histone proteins were used for Western blotting.

Acrolein‐induced neutrophil, H3F3B and PPBP protein increases may represent lung responses to counter suppression of antibacterial immune defense by acrolein. PPBP, also known as CXCL7, induces neutrophil chemotaxis and activation (Bdeir et al., 2016). Histones and histone‐derived peptides display anti‐bacterial activity (Hirsch, 1958; Kim et al., 1996; Zhdan‐Pushkina & Dronova, 1976). Histones are components of neutrophil extracellular traps (NETs) that bind Gram‐positive and ‐negative bacteria and function in bacterial sequestration and killing (Brinkmann et al., 2004). However, the increased neutrophil, H3F3B and PPBP protein may be double‐edged because it can be pathologic. Although lung injury can occur in neutropenic patients (Maunder et al., 1986; Ognibene et al., 1986) and recruitment of active neutrophils to the air space may not cause a significant change in permeability (Martin et al., 1989), excessive infiltrating neutrophils can augment injury (Grommes & Soehnlein, 2010; Potey et al., 2018). Also, available data indicate that circulating extracellular histones can be major mediators of death in liver malfunction and sepsis (Cheng et al., 2019; Xu, Zhang, Monestier, 2011; Xu, Zhang, Pelayo, et al., 2009). Circulating histone H3 level has been associated with fatal outcomes in septic patients (Xu, Zhang, Pelayo, et al., 2009). Deletion of PPBP and PF4 genes that encode the two most abundant platelet chemokines protected mice against acid‐induced ALI pathogenesis (Bdeir et al., 2016). Acrolein induced histone H3 citrullination and NETs formation in a co‐culture of neutrophils and HepG2 cells (Arumugam et al., 2018).

Animal studies and clinical findings suggest acrolein‐induced platelet activation can lead to thrombosis (Sithu et al., 2010; Zirak et al., 2019). The delayed neutrophil and NETs protein increases in acrolein‐exposed mice were followed by or associated with lethality timing. Acrolein‐induced H3F3B and PPBP protein may thus contribute to the progression and development of acrolein‐induced ALI. Because acrolein increased critical components of NETs, that is, total protein, neutrophils, H3F3B, and the platelet protein PPBP, acrolein may induce NETs and or clot formation.

One limitation of this study is that the estrus cycles of females were not synced. Previously, we reported that in the development of sex‐specific silica‐induced fibrosis the response of ovariectomized mice was similar with that of males compared to wild‐type females (Latoche et al., 2016). To examine the role of sex hormones and their receptors in acrolein‐induced lung injury use of ovariectomized mice would be informative.

In conclusion, a better understanding of the underlying mechanisms that govern ALI development and progression may help to develop therapeutic drugs to treat ALI or counter its progression. Acrolein‐induced ALI progression was investigated in differentially sensitive female and male C57BL/6J mice. ALI progression severity was marked by increased BAL total protein associated with increased H3F3B and PPBP lung transcripts and H3F3B and PPBP BAL and plasma proteins. The molecular basis of acrolein‐induced H3F3B and PPBP transcript changes has not been investigated. If transcriptional regulation mediates the acrolein exposure‐induced transcript changes, more investigation is needed to determine the responsible cell type for PPBP transcript change.

## CONFLICT OF INTEREST

None declared.

## AUTHOR CONTRIBUTIONS

Kiflai Bein and George D. Leikauf were responsible for the conception and design of the study. Kiflai Bein, Rahel L. Birru, Heather Wells, Theodore P. Larkin, Tengziyi Ge, and George D. Leikauf were responsible for the acquisition, analyses, and interpretation in this study. Kiflai Bein and George D. Leikauf were responsible for drafting the manuscript for important intellectual content.

## DATA AVAILABILITY STATEMENT

The data that support the findings of this study are available on request from the corresponding author.

## Supporting information



**Figure S1**.

**Table S1**.

**Table S2**.

**Table S3**.

**Table S4**.

**Table S5**.

**Table S6**.

**Table S7**.

**Table S8**.

**Table S9**.

**Table S10**.

**Table S11**.
