# Supplementary material for: Sex‐dependent acrolein sensitivity in mice is associated with differential lung cell, protein, and transcript changes
Source: Physiol Rep. 2021 Oct 4;9(19):e14997. doi: 10.14814/phy2.14997 (PMC8488558; doi:10.14814/phy2.14997)
Supplement: Supplementary file 1 — Figure S1. [file PHY2-9-e14997-s001.docx]

**Supplemental Figure 1. Acrolein exposure of C57BL/6J mice increased BAL and plasma H3.3 histone B (H3F3B) and Pro-Platelet Basic Protein (PPBP) proteins**. Female mice were exposed to 75 ppm acrolein for 30 min and returned to filtered air. (**A**) Bronchoalveolar lavage (BAL) and (**B**) plasma were collected 24 h after exposure. H3F3B and PPBP (25 µl/lane BAL and 10 µl/lane plasma) and haptoglobin, hemopexin, immunoglobulin G (IgG), and albumin (10 µl/lane BAL and 0.5 µl/lane plasma) protein in control and acrolein exposed samples were analyzed by Western blotting. H3F3B and PPBP proteins increased in BAL and plasma from acrolein exposed mice compared with control mice. In contrast, albumin, IgG, and hemopexin increased and haptoglobin decreased in BAL from acrolein exposed mice compared with control mice whereas albumin, IgG and hemopexin slightly decreased in acrolein exposed plasma samples. These results indicate that the increased BAL H3F3B and PPBP protein levels are due to induction by acrolein exposure and not simply due to higher protein concentration because of increased permeability into the alveoli due to respiratory barrier dysfunction.
